# Higher densities of T-lymphocytes in the subsynovial connective tissue of people with carpal tunnel syndrome

**DOI:** 10.1371/journal.pone.0300046

**Published:** 2024-03-07

**Authors:** Oliver Sandy-Hindmarch, Miguel Molina-Alvarez, Akira Wiberg, Dominic Furniss, Annina B. Schmid

**Affiliations:** 1 Nuffield Department of Clinical Neurosciences, University of Oxford, Oxford, United Kingdom; 2 Department of Basic Health Sciences, Area of Pharmacology, Nutrition and Bromatology, Universidad Rey Juan Carlos, Madrid, Spain; 3 Nuffield Department of Orthopaedics, Rheumatology, and Musculoskeletal Sciences, University of Oxford, Oxford, United Kingdom; Istanbul Health and Technology University, Faculty of Medicine, TURKEY

## Abstract

Symptoms in people with carpal tunnel syndrome (CTS) are traditionally attributed to neural tissue, but recent studies suggest that the subsynovial connective tissue (SSCT) may also play a role in CTS. The SSCT undergoes fibrotic thickening which is generally described as “non-inflammatory” based on basic histology. This study uses immunohistochemistry to determine the presence of macrophages and T-cells within SSCT and their relationship with symptoms in people with CTS. SSCT was collected from twenty people with CTS and eight controls undergoing wrist fracture surgery. Immunohistochemical quantification of CD3+ T-cells and CD68+ macrophage densities as well as CD4+/CD8+ T-cell subpopulations were compared between groups using independent t-tests. Spearman correlations were used to identify associations between immune cell densities and CTS symptom scores. The density of CD3+ T-cells was significantly higher in SSCT of people with CTS compared to controls (CTS mean 26.7 (SD 13.7); controls 6.78 (6.3), p = 0.0005) while the density of CD68+ macrophages was lower (CTS mean 9.5 (SD 6.0); controls 17.7 (8.2), p = 0.0058). Neither CD68+ nor CD3+ cell densities correlated with symptom scores. In contrast to previous assumptions, our data show that the SSCT in the carpal tunnel in both people with CTS and controls is not devoid of immune cells. Whereas the higher density of CD68+ macrophages in control participants may be associated with their early recruitment after acute fracture, CD3+ cells within the SSCT may play a role in chronic CTS.

## Introduction

Carpal Tunnel Syndrome (CTS) is an entrapment neuropathy caused by the compression of the median nerve within the carpal tunnel [1]. CTS is characterised by symptoms such as pain, paraesthesia, dysaesthesia, as well as sensory loss in the median-innervated digits, and can progress to thenar muscle weakness and atrophy in advanced stages [2]. Whereas CTS symptoms are traditionally attributed to impairment in nerve function, recent studies suggest that the tendons and associated subsynovial connective tissue (SSCT) also plays a role in CTS pathology [3]. The SSCT comprises connective tissues that surround the median nerve and flexor tendons within the carpal tunnel, and is believed to provide a gliding surface for these structures [4]. It is well established that the SSCT undergoes fibrotic thickening in people with CTS [5–7]. Fibrosis is believed to contribute to median nerve tethering [8], with potential downstream effects such as intraneural ischaemia. The fibrotic changes have repeatedly been described as “non-inflammatory”, as the SSCT is reported to be devoid of immune cells [5, 6, 9]. For instance, Schuind et al [10] found hyperplasia, increased collagen and necrotic regions in the SSCT, but no evidence of inflammation. Similar histological analysis by Gross, et al [11]; Jafari et al [12] and Fuchs et al [13] found necrobiosis of collagen, fibrous thickening of the vasculature and vascular sclerosis respectively in SSCT. In all studies, these changes were determined to be non-inflammatory, with inflammation only detected in a small number of cases (10–11%) [11–14]. As such, inflammation is not considered a cause or a defining feature in the pathological changes of SSCT in people with CTS.

Of note though, the majority of available studies used basic histological staining protocols (e.g., haematoxylin and eosin, H&E) to determine the presence of immune cells in SSCT. This method relies on the morphology of cells instead of their antigenicity. As H&E staining does not directly discriminate different cell types, the determination of immune cells in the tissue is subjective, and observer dependent. To our knowledge, only one study [15] used specific immune cell markers in SSCT (e.g., anti-CD3 for T-cells). This study also reported no significant difference in immune cell presence between people with CTS and a control population consisting of distal radius fracture and flexor tendon injury. However, cell density was not quantified—the presence of inflammation was graded subjectively as absent, mild, moderate or severe.

The notion of non-inflammatory changes in the SSCT is at odds with growing evidence for a role of inflammation in the pathomechanism and symptomatology of people with CTS. Recent studies have found dysregulation in systemic inflammatory mediators between people with CTS and controls [16], as well as within people with CTS pre to post-surgery [17]. In addition, corticosteroid injection, a powerful anti-inflammatory, immunosuppressive and anti-mitogenic agent [18] has short term benefit for people with CTS [19]. These findings, together with improvements in immunohistochemical methodologies and suggestions that CTS symptoms not only arise from alterations in neural but also connective tissue [20], warrant a revisit of the potential presence of SSCT inflammation in CTS and its association with symptoms. In this study we therefore hypothesise that immunohistochemistry with specific immune cell markers will identify increased immune cell densities in SSCT of people with CTS versus control participants. Further, we examine associations of SSCT immune cell densities with patients’ symptomatology. We thereby focus on T-cells and macrophages, two of the major players in peripheral nerve compression [21–23].

## Material and methods

### Study design and participants

This study was part of an existing prospective longitudinal cohort study previously described (recruitment start 18/07/2014, end 18/05/2021) [24]. Twenty people with clinically and electrodiagnostically confirmed CTS were recruited from surgical waiting lists at Oxford University Hospitals NHS Foundation Trust. Exclusion criteria included electrodiagnostic testing revealing an upper limb nerve dysfunction other than CTS, another medical condition affecting the upper limb or neck (e.g. hand osteoarthritis, cervical radiculopathy) or a history of trauma to the upper limb or neck (e.g., previous forearm fractures), coexisting systemic disease (e.g. rheumatoid arthritis, diabetes), or pregnancy. Eight control participants with distal radius fracture requiring open reduction internal fixation were recruited from emergency surgical waiting lists at Oxford University Hospitals NHS Foundation Trust. Control participants with systemic diseases (e.g., diabetes) or a pre-fracture history of medical conditions relating to the upper limb or neck including CTS were excluded. Informed written consent was obtained from all participants and ethical approval was given for the project (Riverside London Ethics Committee Ref 10/H0706/35).

### Phenotypic data of CTS cohort

A detailed description of the phenotypic data collected can be found elsewhere [24]. Age, sex, and duration of symptoms were recorded for each patient. Symptom severity was evaluated with the Boston carpal tunnel questionnaire [25], which contains a symptom and function subscore (0 no symptoms/functional deficit, 5 severe symptoms/functional deficit). Neuropathic pain severity was evaluated with the Neuropathic Pain Symptom Inventory [26] (NPSI), which includes numerical rating scales (0 no pain to 10 worst pain imaginable) for burning, deep pressure, evoked and paroxysmal pain, and paraesthesia as well as a composite score (0–100). Severity of pain over the past 24 hours was recorded on a visual analogue scale (VAS; 0 no pain, 10 worst pain imaginable). Standard electrodiagnostic testing of the median, ulnar and superficial radial nerve was performed with an ADVANCE^™^ system (Neurometrix, USA). Median nerve electrodiagnostic test severity was graded as follows [27]: normal (grade 0), very mild (grade 1), mild (grade 2), moderate (grade 3), severe (grade 4), very severe (grade 5), extremely severe (grade 6). Radial and ulnar nerve data were used to exclude other upper limb neuropathies. Further details of electrodiagnostic test methodology can be found in our previous work [28].

### Tissue collection and preparation

SSCT from the flexor tendons was surgically resected during CTS decompression surgery for people with CTS. As ethical reasons preclude the collection of SSCT from healthy people, we collected control SSCT during open reduction and internal fixation surgery for distal radius fractures (volar plates). Whereas SSCT from people with CTS were harvested inside the carpal tunnel, it was taken more proximally but as far distally as the incision allowed in control participants.

Samples were immediately placed into fresh periodate-lysine-paraformaldehyde (PLP) fixative. Samples remained in PLP fixative at room temperature for 6hrs before being washed 3x with 0.1M phosphate buffer. Washed samples were placed in 4.4M sucrose solution and stored at 4°C for 42–74 hrs. Samples were then frozen in optimal cutting temperature (OCT) in base moulds and stored at -80°C.

### Immunohistochemistry

Immunohistochemistry was performed on the SSCT samples from patients and controls to detect specific immune cell densities. For each sample, two serial 14μm sections were cut using a cryostat (Leica, Germany) and adhered to separate gelatinised glass microscope slides (Thermo Fisher Scientific, UK). Slides were gelatinized by dissolving 1.5g Gelatine type A (Sigma Aldrich, UK) in 500ml of 60°C dH_2_O. Once the gelatine had dissolved fully, 0.25g of chromium potassium sulphate was added. Slides were dipped into warm gelatine solution (40°C– 50°C) twice, left to drain overnight and then baked for 1 hr at 60°C to improve section adherence. Mild Heat Induced Epitope Retrieval (HIER) was done by incubating slides in EDTA buffer (10mM tris Base, 1mM EDTA, 0.05% Tween, pH9.0) for 4-5hrs, a method adapted from Dawes et al [29]. After HIER treatment, slides were washed for 3 minutes in PBS. Sections were incubated in blocking solution (PBS + 0.2% TritonX + 10% goat serum) for 1hr before the application of the primary antibody. To each slide, either a rabbit anti-human CD3 (Abcam, 1:100, cat no: ab16669) or a rabbit anti-human CD68 (Abcam, 1:200, cat no: ab213363) was added and incubated overnight at 4°C in a humidified chamber. The next day, slides were washed 3x for 10 minutes each in PBS + 0.2% Triton X and an Alexa Fluor 546 anti-rabbit secondary antibody (Thermo-fisher, 1:500, cat no: A10040) was applied. Sections with secondary antibodies were incubated at room temperature in a dark humidified chamber for 2 hours. After incubation, slides were washed three times for 10 minutes in PBS + 0.2% Triton X before sections were covered with mounting media containing DAPI and a coverslip. Sections were imaged as maximum intensity projections of Z-stacks at 20x magnification on an Observer Z1 Confocal imaging system (Zeiss, Germany). Full sections were imaged unless the samples were larger than 9mm^2^, in which case six randomly selected images of 1.5mm^2^ were taken to cover a significant proportion of the section.

### Cell counting

Imaging and counting were performed by an examiner blinded to group allocation. In each image, positively stained immune cells with a DAPI positive nucleus were manually counted using the counting tool in ImageJ. The counted tissue area was measured in ImageJ. The number of cells was then divided by the tissue area and the tissue thickness for each section to give a cell density in cells/mm^3^. The cell densities for the images of each section were then averaged to give average cell densities for each cell type in each patient.

### Statistical analysis and sample size

Sample size was based on our pilot CD3 data in SSCT of n = 19 people with CTS grouped as mild (Boston carpal tunnel symptom questionnaire of ≤1) or severe symptom severity (Boston carpal tunnel questionnaire of ≥4). Assuming that control SSCT immune cell infiltration is comparable with those of patients with mild symptom severity, n = 20 people with CTS and n = 8 controls were required to identify significant group differences (two-tailed independent t-test, allocation ratio 0.4, d = 1.25, 80% power, α = 0.05).

All statistical analyses were conducted using SPSS (version 28, IBM). Visual inspection of the data, as well as Shapiro Wilk tests were used to test for normality. To determine differences in immune cell density between people with CTS and controls, independent samples t-tests were conducted with P<0.05 being significant. All correlations were conducted using the Spearman’s rank correlation to account for non-parametric questionnaire data. P<0.05 was set as the significance threshold throughout.

## Results

Clinical characteristics of patients and controls can be found in Table 1. Patients had chronic CTS symptoms of moderate severity. People with distal radius fracture were usually operated at a median of 2 days (interquartile range 5 days) following injury.

**Table 1:** Clinical characteristics. Data are presented as median with [interquartile range] unless indicated otherwise.

	Controls	CTS
Number of Participants	8	20
Age, mean years (SD)	53 (17.53)	63 (11.66)
Female gender, n (%)	5 (62.50)	14 (70)
Height (cm)	169 [26.50]	168 [10.92]
Weight (kg)	75.50 [31.75]	67.00 [15.25]
BMI (kg/m^2^)	26.03 [4.90]	24.16 [4.21]
Duration of symptoms (months)		30 [81]
electrodiagnostic test grade		3.5 [2.75]
Normal, n		0
Very mild, n		2
Mild, n		3
Moderate, n		5
Severe, n		1
Very severe, n		7
Extremely severe, n		2
Boston symptom score		2.84 [1.07]
Boston function score		2.44 [1.00]
VAS pain		3.00 [4.05]
NPSI total score		10.42 [17.75]
burning pain		0.0 [6.0]
deep pain		2.0 [3.6]
evoked pain		1.0 [3.4]
paraesthesia		4.0 [4.8]
paroxysmal pain		1.5 [3.4]

**Fig 1:**
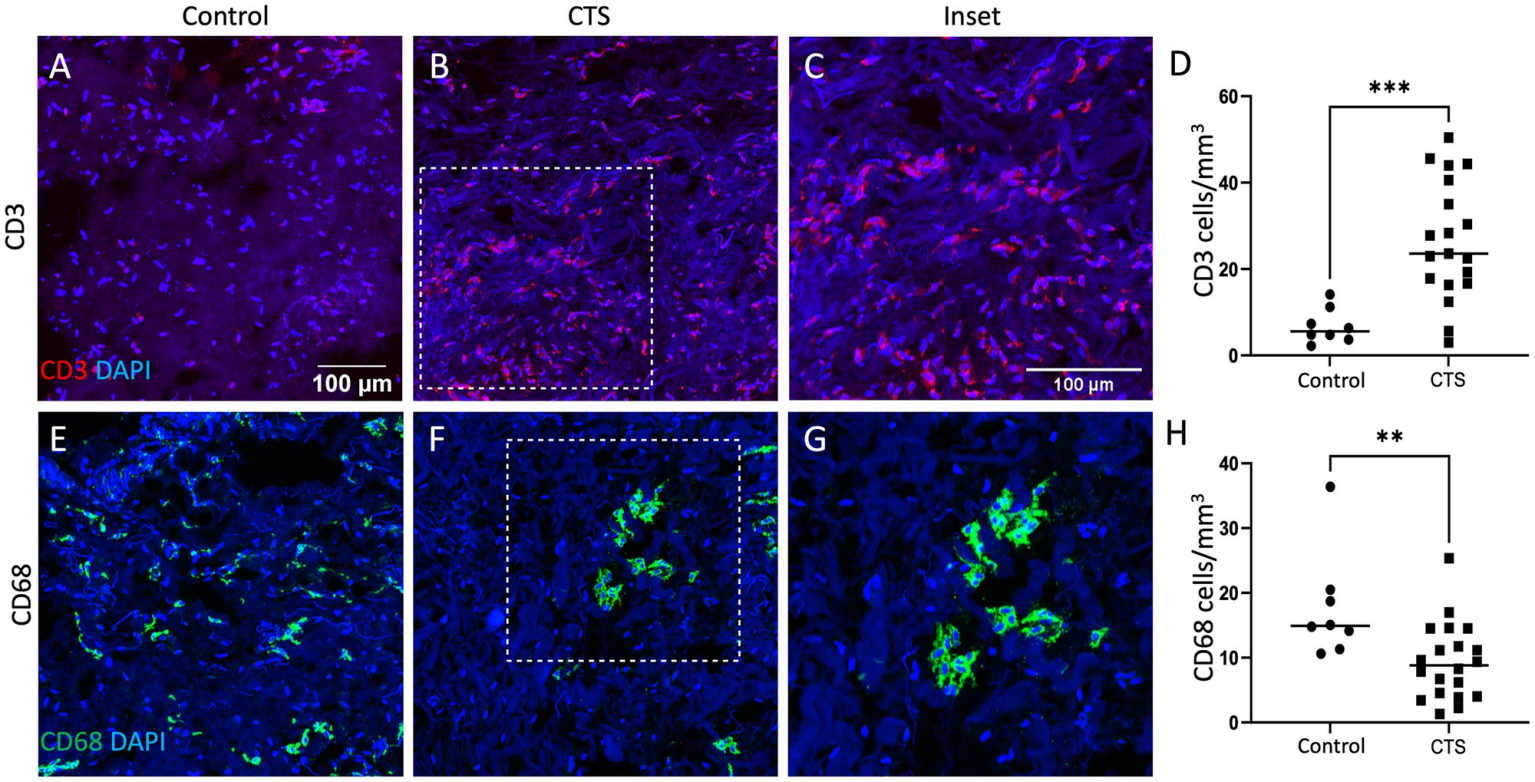
CD3+ T-cells and CD68+ cells are present in subsynovial connective tissue (SSCT) and significantly differ between people with CTS and controls. Immunohistochemically stained sections of SSCT from controls and people with CTS. Cell nuclei are stained with DAPI and are shown in blue. T-cells were identified by CD3 (red; A-C). T-cells densities are higher in tissue of people with CTS (B inset enlarged in C) compared to controls (A). This is quantified in (D). CD68 is a lysosomal protein that is enriched in macrophages (green; D-F). CD68^+^ cell density was higher in SSCT of controls (E) compared to people with CTS (F, inset enlarged in G). This is quantified in (H). independent samples t-test, **P = 0.006, ***P<0.001.

The density of CD3+ T-cells was significantly higher in SSCT of people with CTS compared to controls (CTS mean 26.7 (SD 13.7); controls 6.8 (4.0), p<0.001, Fig 1A–1D). In contrast, the density of CD68+ cells was lower in SSCT of people with CTS compared to controls (CTS mean 9.4 (SD 5.9); controls 17.7 (8.2), p = 0.006, Fig 1E–1H). Neither CD68+ nor CD3+ cell densities correlated with symptom scores (p>0.12, S1 Table).

## Discussion

Immunohistochemical analysis of SSCT from people with CTS and controls revealed the presence of both macrophages and T-cells in the tissue, with CD3 and CD68 cell markers detectable. CD68+ cell densities were significantly lower in SSCT from people with CTS compared to control tissue, while CD3+ T-cell densities were significantly higher in SSCT of people with CTS compared to controls. No significant correlations were found between CD3+ and CD68+ cell densities and clinical symptom scores.

The significantly higher density of CD3+ T-cells in the SSCT of people with CTS indicates the presence of T-cell mediated low-grade inflammation within the tissue. This is in contrast to several immunohistochemical studies conducted in the SSCT of people with CTS, which found inflammation to be either not present [5, 10] or present in very few cases [11–13]. For instance, Fuchs et al [13] and Nakamichi et al [14] reported the presence of inflammatory cells in SSCT in only 10% of people with CTS based on H&E staining. Using their definition, inflammatory cells were detected in 100% of the samples tested in our study, which is remarkably higher. This discrepancy is likely due to the use of antibodies specific for immune cell types in our study, rather than nonspecific H&E staining. By using these antibodies, we have been able to confirm the presence of immune cells by quantification of specific molecular markers as opposed to only morphological features, which relies on subjective judgement and is thus prone to bias. The only study to date also to have used specific immune cell markers in SSCT of people with CTS reported that only 16% of CTS samples and 7.1% of control samples had CD3+ T-cell infiltration [15]. In our study, CD3 positive T-cells were present in 100% of CTS and control samples. This discrepancy could be explained by the differences in staining protocols. We used several rounds of optimization to generate IHC stains of sufficient quality. Importantly, we found that the use of HIER was essential to unmask antigens. Indeed, had we not persevered with antibody optimization, we would have substantially underestimated the number of immune cells present in tissues due to poor signal to noise ratio. Unfortunately, it is difficult to compare our staining techniques to the Yeşil study [30] as no detail is provided on their immunohistochemistry protocol and no images of CD3 staining are available.

Our study design does not allow us to infer whether the higher density of T-cells is causative and/or an epiphenomenon of the compression. Indeed, chronic mild experimental nerve compression induces inflammatory cell recruitment both intraneurally as well as in extraneural connective tissues [23, 31]. T-cells have been implicated in fibrosis [32], which in the context of the SSCT is associated with tethering of the median nerve [8] and potential downstream effects such as intraneural ischaemia, resulting in nerve dysfunction and neuropathic pain. Additionally, T-cells may elicit pain through direct effects on the SSCT. While we could not find literature on innervation of SSCT in humans, other human synovial connective tissue, for instance from wrist joints, are innervated with Substance P positive (SP^+^) nerve fibres indicative of nociceptive innervation [33]. A local inflammatory environment within the SSCT might contribute to their sensitization and explain why a subset of patients experience nociceptive rather than neuropathic pain [34]. This hypothesis is in agreement with the suggestion by Hirata et al [20] that some symptoms of CTS are caused by alterations to connective tissue instead of nerve pathology.

Unexpectedly, CD68^+^ cell density was significantly lower in SSCT of people with CTS compared to controls. This may appear contradictory to the finding of higher T-cell density. Whereas this differential regulation may reflect differences in immune cell type participation, it may also reflect their distinct temporal patterns. The median symptom duration for people with CTS was 30 months whereas the control participants were operated on within a median of 2 days after radius fracture. Quite likely, the recent fracture initiated an acute inflammatory response, which is expected to be characterized predominantly by macrophages at these early time points [35]. T-cell recruitment usually occurs only later after injury–in outer tendon tissues they peak around 2–3 weeks after injury [36]–but are known to persist in tissues long after acute injury. As such, the SSCT from people with recent distal radius fracture, albeit one of the few options to sample non-CTS SSCT, may not be a perfect control tissue, particularly for cells involved in early immune dysregulation. Nevertheless, the higher CD3+ T-cells density in people with CTS in the chronic stage is intriguing and may suggest that CTS is associated with persistent chronic T-cell driven low-grade inflammation, a phenomenon seen in other musculoskeletal conditions such as rheumatoid arthritis [37] and tendinopathies [38]. While CD68 is commonly used as a marker for macrophages, non-myeloid cells such as fibroblasts or endothelial cells may show immunoreactivity to some CD68 clones [39]. Therefore, our finding could also represent the loss of non-myeloid CD68+ cells such as fibroblasts. However, we believe this is unlikely given previous reports of marked increases of fibroblast densities in SSCT of patients with CTS [40].

This study has a number of limitations. First, participants in the control group had trauma to the distal radius as it was not ethically possible to obtain SSCT from healthy people. We decided against using post-mortem tissues due to the challenges of a) delayed/altered tissue processing influencing immunostaining [41–43], b) age matching (e.g., immune cell senescence) [44] and c) obtaining accurate medical histories to assure the exclusion of CTS and diseases influencing the immune system. As discussed above, it is possible that the trauma to the forearm, although in a different area to the wrist, has caused a general increase in infiltrating immune cells and inflammation, making it more challenging to identify differential immune cell densities between groups. Further, sampling locations were different, with SSCT from people with CTS taken within the carpal tunnel and slightly more proximal in people undergoing internal fixation surgery. While potential histological differences in SSCT location may have influenced our findings, this would unlikely explain the opposing effect on CD68+ cells and T-cells. In addition, we were limited in the degree to which we could phenotype the immune cells within the tissue due to sample shortage-a common problem when working with human tissues. Macrophages and T-cells include diverse subsets with varying roles in tissue destruction and repair as well as pain maintenance and its resolution [45, 46]. Future studies are required to identify the specific subtypes of immune cells in the SSCT of people with CTS.

## Conclusions

To conclude, this study conclusively shows that the SSCT in the carpal tunnel is not devoid of immune cells; this contrasts with common beliefs. We were able to demonstrate a significantly higher density of CD3+ T-cells within the SSCT of people with CTS compared to controls. CD68+ cell densities were lower in SSCT from people with CTS compared to SCCT from acute fracture patients. This may be due to temporal patterns of macrophage involvement being more prominent in an acute fracture scenario compared to chronic CTS, or it could indicate the loss of other CD68+ cell types such as fibroblasts in persistent CTS. Even though no correlations were found between different immune cells and clinical symptomatology, our results suggest the involvement of immune cells and low-grade inflammation, particularly of CD3+ cells in CTS, thus challenging the widely held notion of a “non-inflammatory fibrosis” of the SSCT as a hallmark of CTS pathophysiology. Our results provide the foundation for future research to further interrogate the role that inflammation, and specifically T-cells may play in the pathogenesis of CTS in extraneural tissues.

## Supporting information

S1 Table Correlations between CD68 or CD3+ cell density and CTS patients’ symptoms. Table depicts p-values for Spearman’s correlations.
(DOCX)
