# Supplementary material for: Higher densities of T-lymphocytes in the subsynovial connective tissue of people with carpal tunnel syndrome
Source: PLoS One. 2024 Mar 7;19(3):e0300046. doi: 10.1371/journal.pone.0300046 (PMC10919581; doi:10.1371/journal.pone.0300046)
Supplement: S1 Table — Table depicts p-values for Spearman’s correlations. (DOCX) [file pone.0300046.s001.docx]

Table S1: Correlations between CD68 or CD3+ cell density and CTS patients’ symptoms. Table depicts p-values for Spearman’s correlations.

|  | CD68 | | CD3 | |
| --- | --- | --- | --- | --- |
|  | r | p-value | r | p-value |
| Boston symptom | 0.27 | 0.24 | 0.37 | 0.12 |
| VAS pain | 0.21 | 0.41 | 0.29 | 0.27 |
| NPSI burning | -0.09 | 0.70 | 0.04 | 0.88 |
| NPSI deep | -0.01 | 0.97 | 0.20 | 0.41 |
| NPSI evoked | 0.05 | 0.83 | 0.15 | 0.55 |
| NPSI paroxysmal | 0.29 | 0.21 | 0.26 | 0.29 |
| NPSI paraesthesia | -0.08 | 0.75 | 0.07 | 0.77 |
| NPSI total | -0.06 | 0.79 | 0.12 | 0.63 |

VAS: visual analogue scale; NPSI: neuropathic pain symptom inventory
